# Effect of Hydrogel Substrate Components on the Stability of Tetracycline Hydrochloride and Swelling Activity against Model Skin Sebum

**DOI:** 10.3390/ijms24032678

**Published:** 2023-01-31

**Authors:** Agnieszka Kostrzębska, Karolina Pączek, Angelika Weselak, Witold Musiał

**Affiliations:** Department of Physical Chemistry and Biophysics, Faculty of Pharmacy, Wroclaw Medical University, Borowska 211A, 55-556 Wroclaw, Poland

**Keywords:** tetracycline hydrochloride, hydrogel, alcoholamine, stability, skin sebum, carbomer, TRIS

## Abstract

Due to its high instability and rapid degradation under adverse conditions, tetracycline hydrochloride (TC) can cause difficulties in the development of an effective but stable formulation for the topical treatment of acne. The aim of the following work was to propose a hydrogel formulation that would ensure the stability of the antibiotic contained in it. Additionally, an important property of the prepared formulations was the activity of the alcoholamines contained in them against the components of the model sebum. This feature may help effectively cleanse the hair follicles in the accumulated sebum layer. A series of formulations with varying proportions of anionic polymer and alcoholamine and containing different polymers have been developed. The stability of tetracycline hydrochloride contained in the hydrogels was evaluated for 28 days by HPLC analysis. Formulations containing a large excess of TRIS alcoholamine led to the rapid degradation of TC from an initial concentration of about 10 µg/mL to about 1 µg/mL after 28 days. At the same time, these formulations showed the highest activity against artificial sebum components. Thanks to appropriately selected proportions of the components, it was possible to develop a formulation that assured the stability of tetracycline for ca. one month, while maintaining formulation activity against the components of model sebum.

## 1. Introduction

Acne vulgaris is a dermatosis affecting more than 80% of young adults [1]. Acne therapy has long been a challenge for medicine. There is no single perfect solution that provides an effective, as well as completely safe, solution for patients. The treatment process is usually prolonged and requires a systematic approach on the part of the patient. There are two main modes of acne treatment, depending on the extent of the problem.

For the most part, the disease has a mild course and topical treatment is usually applied by relieving inflammation, regulating excessive keratinization of the skin or inhibiting the growth of bacteria responsible for the development of the disease. However, about 15–20% of patients develop a severe form of the disease, and it is necessary to implement systemic treatment. The main groups of drugs used in oral treatment are antibiotics, retinoids and hormonal preparations [2,3,4].

Over half of patients can be successfully cured with topical treatments without the need to introduce systemic therapies which carry a number of side effects. Oral acne management should be a last resort, so it is vitally important to constantly search for the most effective topical treatments.

In addition to the proper selection of effective antimicrobial preparations, a significant difficulty in acne therapy is the successful penetration of the drug into the sebum-filled interior of the hair follicle. In clogged hair follicles, there is an intense proliferation of microorganisms and an increase in inflammatory processes. Good and effective penetration of the therapeutic preparation deep into the hair follicle can provide stronger, more effective and shorter therapy. 

The hydrogel formulations presented in this paper are a proposal for preparations with a potential multifaceted course of action, involving the cleansing of the hair follicle area to allow substances with antimicrobial properties to effectively penetrate into the sebaceous hair unit. 

Tetracycline hydrochloride was chosen as the antimicrobial ingredient for the formulations. Tetracyclines are one of the main groups of antibiotics used to treat acne [5,6,7,8]. Tetracycline is a broad-spectrum antibiotic that has been widely used in the treatment of acne in both oral and topical forms. In addition to its bacterial inhibitory effect, topical tetracycline has nonantibiotic effects, such as inhibiting inflammatory processes. In addition, it can inhibit pathological angiogenesis, which accompanies inflammatory processes [9]. These are valuable properties that can enhance the topical treatment of bacterial infections and associated inflammation. At the same time, antibiotics of the tetracycline group are unstable substances, highly sensitive to adverse external conditions. They decompose when exposed to excessive temperature, moisture or exposure to light. Additionally, unfavourable pH values for tetracyclines—either excessively acidic or excessively alkaline—lead to their breakdown [10,11,12,13,14]. As a result of the transformations that occur, these antibiotics often lose their antibacterial properties. In addition, the degradation products may exhibit toxic phototoxic or nephrotoxic properties [11,12,15,16,17].

In our previous studies, we have analysed the effects of varying environmental temperature and photoprotection or its absence on TC stability. Antibiotic hydrogel formulations and their corresponding aqueous solutions of TC were evaluated regarding the effect of external conditions. We proved that TC shows significantly higher stability in hydrogels than in aqueous solutions. Moreover, appropriate storage conditions for the formulation, such as low temperature and photoprotection conditions, significantly inhibit the degradation of tetracycline hydrochloride [18].

The natural sequence, then, was to evaluate how the stability of tetracycline hydrochloride is affected by the varying composition of hydrogel formulations. 

The second phenomenon evaluated in this study was the activity of the formulations against model sebum components. Proliferating within the hair follicles, the pathological skin microbiome causes local inflammation with painful skin lesions. Lingering sebum in the hair follicles effectively inhibits therapeutic agents aimed at eliminating bacteria and inhibiting inflammation from reaching these areas. Previous studies showed that alcoholamines react with model sebum components such as stearic acid, leading to saponification and the appearance of a light layer of products on the sebum surface (Equation (1)) [19,20,21,22].

R_1_—COOH + R_2_—NH_2_ (aq) → R_1_—COO^−^[NH_3_^+^—R_2_] (aq) (1)

The hydroxyl groups present in the molecules of alcoholamines can bind to water, so the resulting layer is loosened. This phenomenon may help cleanse the hair follicles of the sebum layers that are lodged in them. This process might simplify the introduction of active therapeutic substances inside the hair follicles and effectively eliminate resident bacteria such as *C. acnes*, *S.aureus* and others. 

The purpose of the present study was to search for mutual proportions of the components of the hydrogel to ensure, on the one hand, the stability of the antibiotic with preserved antibacterial activity, and on the other hand, to not inhibit the reactivity of the alcoholamine contained in it against the components of the model sebum.

This may help in the future to develop an effective multidirectional therapy for acne, combining the antibacterial activity of the antibiotic with the hair follicle—cleansing effect of alcoholamines. 

An important aspect of the presented research is the exploration of more effective methods of delivering therapeutic agents deep into the hair follicle. Our proposed method of reaching the interior of the hair follicle by saponification and forming an emulsion that loosens sebum deposits is, in our opinion, worthy of consideration. 

## 2. Results

### 2.1. pH Measurements of Formulations

Acrylic acid polymer neutralized with 2–amino–2–(hydroxymethyl)–1,3–propanediol alcoholamine (TRIS) was used to prepare the formulation. The variable ingredient in the tested formulations was the acrylic acid polymer. Two series of hydrogels were prepared. The first series contained a constant TRIS amount and a variable carbomer content. This procedure resulted in four hydrogels, 1–4, where gel 1 with the lowest carbomer content showed the highest pH value of 8.36. Formulation 2 at the higher polymer content had a pH value of 8.04. Formulations 3 and 4 with increased polymer content achieved increasingly lower pH values of 7.60 and 5.00, respectively. The second series consisted of three A–C formulations, which were prepared with three different carbomers to evaluate whether the type of acrylic acid polymer affects the durability of tetracycline hydrochloride. They had a similar slightly acidic pHs of about 6.34. The pH value for each sample was measured five times just after hydrogel preparation. The results are shown in Table 1.

### 2.2. Assessment of TC Stability by HPLC Analysis

The stability of the antibiotic was assessed by HPLC analysis at specified intervals for a period of 28 days. Two series of hydrogels were designed. In series 1–4, the effect on the stability of the drug was evaluated by changing the ratio of polymer to alkaline alcoholamine. In series A–C, it was assessed how the type of acrylic acid polymer affects the stability of tetracycline hydrogels.

**Figure 1:**
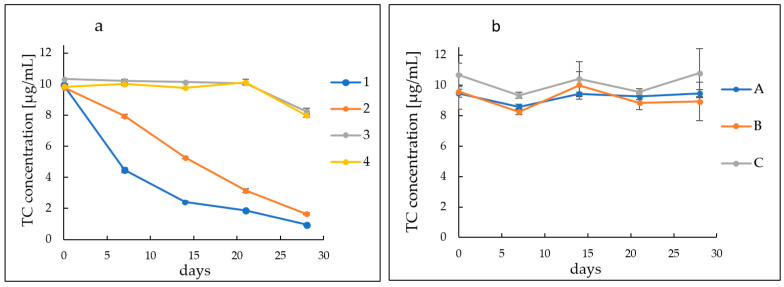
Course of TC concentration changes in hydrogels. (**a**) Formulations (1–4) with variable polymer content and (**b**) formulations (A–C) with variable polymer type.

Both groups of hydrogel preparations were subjected to HPLC analysis lasting 28 days. The content of antibiotics in all developed gels was 0.2 g/100 g. In the case of the first group with variable formulation pH values, high TC instability and progressive degradation were observed for some hydrogels. Samples 1 and 2 had high pH values of 8.36 and 8.04, respectively. These samples showed a gradual decrease in TC values over time. In the case of sample 1, the degradation of tetracycline is strongly noticeable. The concentration of the drug in this preparation decreased from a value of 9.92 µg/mL on the first day of measurements to 4.47 µg/mL on the seventh. On the 14th day of measurements, the concentration reached a value of 2.4 µg/mL, to achieve a value of 0.96 µg/mL on day 28. For sample 2, the drug concentration reached 7.95 µg/mL on the 7th day. On the 14th day of observation, the tetracycline concentration reached 5.27 µg/mL, while on the last day it became 1.65 µg/mL. The remaining samples, both 3 and 4 and A–C, showed high stability of the antibiotic for 28 days of observation. Concentrations of TC oscillated around the initial values of the drug. For samples 3 and 4, the initial concentrations were about 10 µg/mL, while on the 28th day of observation they were approximately 8 µg/mL. For sample A, the starting and ending concentrations were around 9.5 µg/mL with slight fluctuations during the observations. The values of sample B changed from an initial state of 9.6 µg/mL to 8.9 µg/mL, while the initial and final values of sample C were similar and oscillated around the initial value of 10.7 µg/mL. The described changes are shown in Figure 1.

### 2.3. Macroscopic Observations of the Colour Changes of the Hydrogels

Tetracycline hydrochloride is a light-yellow coloured substance. All of the tested preparations were bright yellow in colour at the beginning of the observations. In the case of hydrogels from group 1–4, a colour change was noticed very quickly for the alkaline samples. In the case of sample 1, the hydrogel turned orange after just 7 days, then it was maroon until a black colour observed on day 28. For sample 2, the colour changes were more gradual. After 7 days, the gel turned dark yellow and became red on day 14. On the 28th day of observation, the gel became dark brown. Samples 3 and 4 showed no significant colour changes throughout the observation period, indicating that the antibiotic remained stable. On the 28th day of observation, the gels maintained their yellow colour.

**Figure 2:**
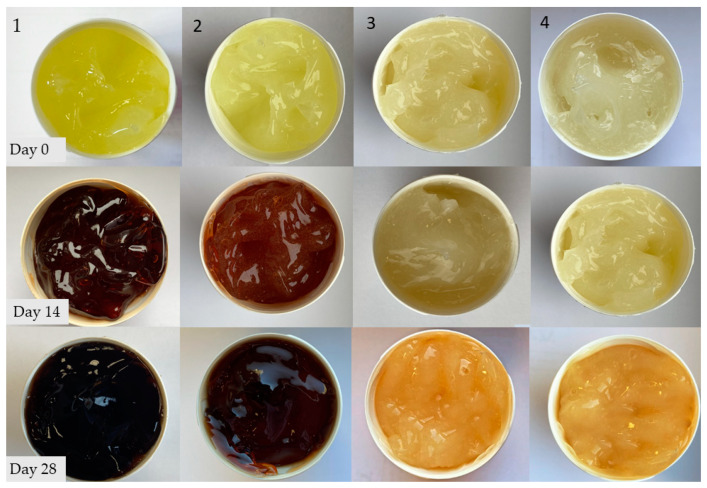
Colour changes of hydrogel preparations (**1**–**4**) noted during the 28-day observation period.

**Figure 3:**
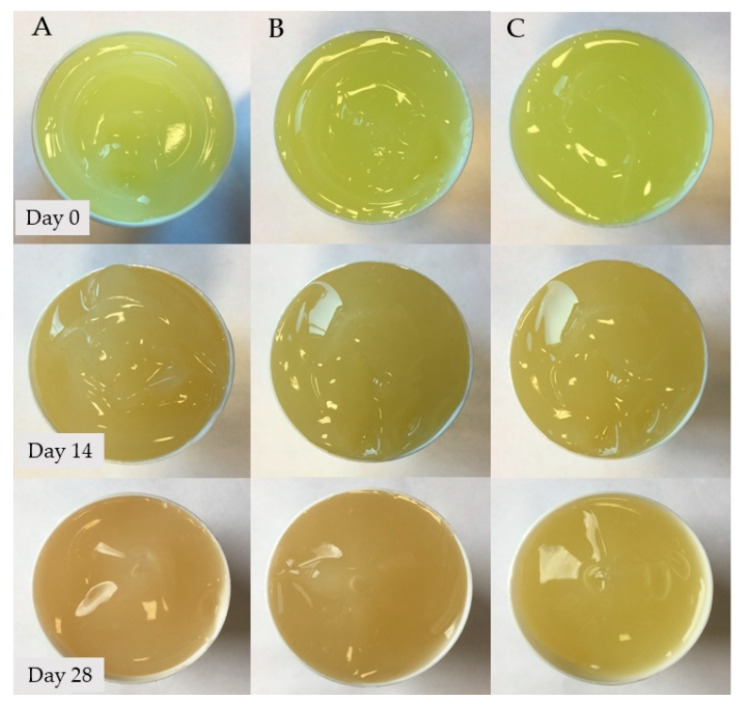
Colour changes of hydrogel preparations (**A**–**C**) noted during the 28-day observation period.

Hydrogels of the A–C group showed no significant colour changes over the entire observation period. The colour changes that occurred in the tested hydrogels are shown in Figure 2 and Figure 3.

### 2.4. Assessment of the Activity of Hydrogels against the Components of Model Sebum

**Figure 4:**
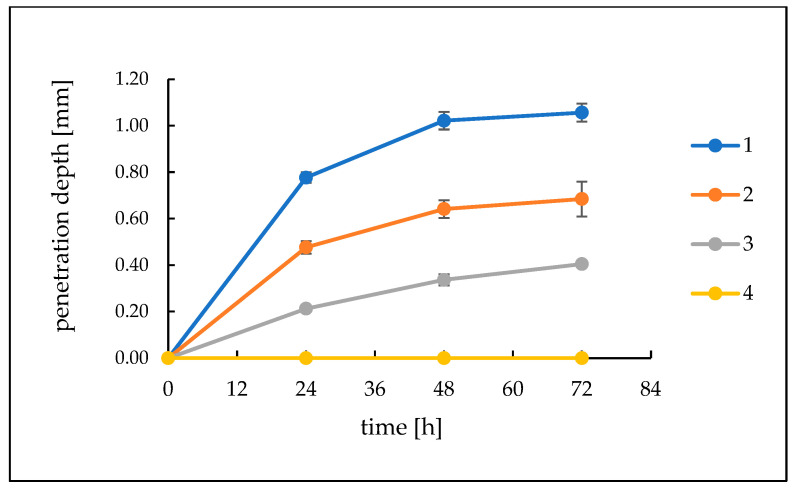
The course of changes in the thickness of the resulting layer of products on the surface of the model sebum over 72 h for Formulations (1–4).

**Figure 5:**
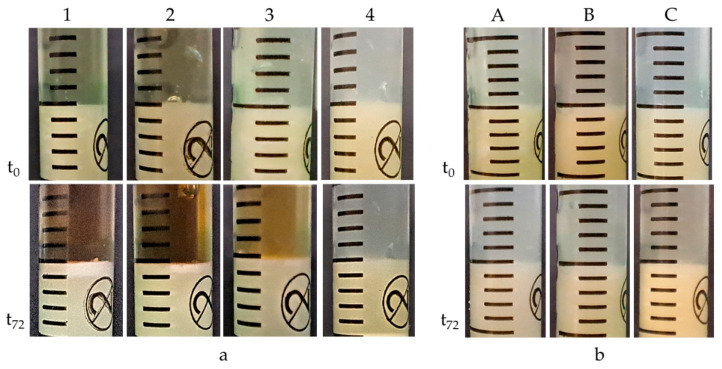
(**a**) Formulations (1–4): visible bright layer of reaction products on the surface of model sebum for samples (1–3) and no reaction for sample 4. (**b**) Formulations (A–C): inactivity of hydrogels against model sebum components.

The hydrogels were analysed for 72 h for their activity against model sebum ingredients. After 24 h, a visible, bright product layer was observed for Formulations 1, 2 and 3. For Formulation 1, with the highest pH value, this layer was the deepest, reaching 0.78 mm. For hydrogel 2, a slightly less deep etching layer appeared, achieving a depth of about 0.48 mm, while for Formulation 3, the layer was very thin, about 0.21 mm in thickness. After another 24 h, the depth of etching increased, reaching values of 1.02 mm for Formulation 1, 0.64 mm for Formulation 2 and 0.34 mm for Formulation 3. After 72 h, the depth of the product layers did not change significantly. For Formulation 4, with a pH value of 5, there was no change in the sebum surface throughout the observation period. Similarly, for A–C preparations with a pH value of around 6.3, no changes were observed on the surface of the model sebum for 72 h of observation. The course of changes on the surface of the model sebum and the appearing product layers are shown in Figure 4 and Figure 5.

### 2.5. Measurement of Viscosity of Hydrogel Formulations

The viscosity evaluations of polymeric formulations, as well as the relative proportions of polymer and amine, gave an insight into the influence of composition on the rheology. Formulations 1–4 had the same TRIS alcoholamine content but differed in the amount of the carbomer used. Formulation 1 had a significant excess of amine, with a ratio of 3:1 to carbomer. This had a noticeable effect on viscosity, the level of which was the lowest compared to others in the batch, reaching a value close to 20,675 cP. In samples 2 and 3, the carbomer content was almost half that of alcoholamine; for sample 2 it was a ratio of 2.25: 1 and for sample 3 the ratio was 1.69: 1, which increased the viscosity, reaching values of 24,737 cP and 28,726 cP, respectively. The formulation with the highest viscosity of 38,274 cP is Formulation 4, in which the carbomer content exceeded that of TRIS, and the polymer-to-amine ratio was 1.37:1. 

**Figure 6:**
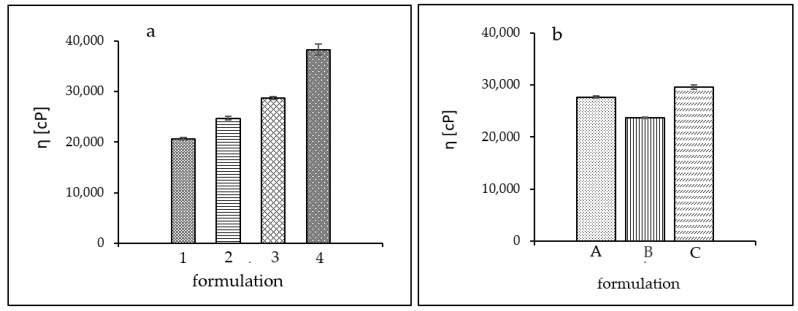
Viscosity values obtained. (**a**) Formulations (1–4) and (**b**) Formulations (A–C).

For Formulations A–C, no significant differences in viscosity values were observed for individual polymers. The selected carbomers had similar viscosities, and the viscosity value for each formulation ranged from 23,696 for B through 27,680 for A to 29,552 for C. The viscosity values obtained are shown in Figure 6.

## 3. Discussion

**Figure 7:**
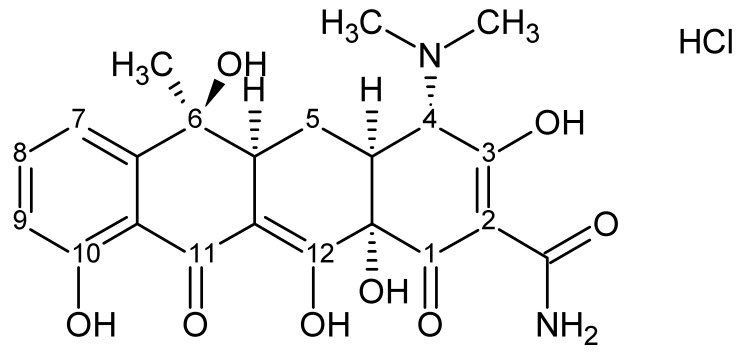
Chemical structure of tetracycline hydrochloride.

Antibiotics from the tetracycline group have been used for years to treat acne, most commonly in the form of oral preparations. These are broad–spectrum antibiotics with a mechanism based on inhibition of bacterial protein synthesis. They exhibit antibacterial activity against Gram–positive, Gram–negative and anaerobic bacteria. They are used in the treatment of bacterial infections of soft tissues and skin caused by strains of *S. aureus*, *S. epidermidis* or anaerobes of the genus *C. acnes* [23,24]. Topically applied tetracycline, in addition to inhibiting bacterial proliferation, has anti-inflammatory effects, which may be a beneficial property for the treatment of acne lesions [9]. At the same time, tetracycline is an unstable substance and very sensitive to external conditions and storage. Figure 7 shows the chemical structure of tetracycline hydrochloride.

Improper storage temperature, high humidity or exposure to light leads to rapid degradation with the formation of numerous breakdown products [13,15,25,26]. Tetracycline is known to be unstable at extreme pH values and degrades rapidly [18,26,27,28]. Frequently, these processes are accompanied by a visible change in colour, i.e., from light yellow to brown and maroon to black, as noted by numerous research teams [14,25,29]. The resulting degradation products usually do not exhibit antimicrobial properties, and sometimes toxic compounds appear. Examples are various photosensitizing derivatives formed by light–induced decomposition of TCs, such as lumitetracycline. There is a significant remodelling of the molecule mainly between C-4-C-11a and the substituents at C–11 and C–12 [13,30]. In a highly acidic environment, at a pH below 2, dehydration occurs at the C–5a–C–6 position and anhydrotetracycline (ATC) is formed, which converts to a 4–epianhydrotetracycline epimer (4–EATC). It exhibits nephrotoxic effects, leading to reversible Fanconi syndrome [10,11,12,17,31]. In a weakly acidic to neutral environment, tetracycline shows high stability. Reversible epimerization then occurs with the formation of epitetracycline (ETC), which has no active antimicrobial activity. According to various researchers, this phenomenon occurs at pH ranges of 3–5 or 2–6 [10,32,33]. Alkaline pH values are undesirable for TC. In an alkaline environment, rapid decomposition of the compound occurs with the formation of a dark maroon, almost black product [18]. Davies et al. and other researchers indicated in their research that the maroon colour may be derived from the resulting quinones. They suggest that in aqueous solutions at highly alkaline pH in the presence of oxygen and irradiation, photodeamination at the C–4 position and loss of the substituent at the C–12a position occurs with the formation of a quinone [11,13,27,28,30]. In our previous studies, we evaluated the effect of varying environmental conditions on TC stability [18]. Tetracycline hydrochloride stored at low temperature and with photoprotection showed significantly higher stability. In addition, the stability of the antibiotic was definitely improved by the hydrogel formulation. In the present study, we investigated how varying the formulation composition would affect the stability of tetracycline hydrochloride. By varying the proportions between the components in gels 1–4, the systems varied significantly in pH values. From strongly alkaline (8.36) through alkaline (8.04) and close to neutral (7.60) to slightly acidic (5.00). The effect of such different pH values on the stability of tetracycline was evaluated by HPLC analysis. The analysis was conducted for 28 days taking samples at equal intervals once a week. For samples with an alkaline pH, a rapid decrease in TC content was observed, reaching a value of 0.96 µg/mL for Formulation 1 and 1.65 µg/mL for hydrogel 2 on day 28. Samples 3 and 4 maintained high stability throughout the study period, reaching concentrations of around 8 µg/mL on the 28th day of measurement. For samples A–C, high TC stability was observed throughout the analysis period, with slight fluctuations in concentrations oscillating around baseline concentrations. These slight fluctuations in sample concentrations during the study may be attributed to small changes in the distribution of the drug in the hydrogel formulation. This may be indicated by SEM analysis performed during previous studies, showing the presence of small aggregates and structures in the hydrogel, suggesting local TC precipitation [18]. HPLC analysis found that despite the preserved external conditions and formulation, which favourably affects the stability of TC, excessively alkaline values of the formulation lead to rapid degradation of the antibiotic. At the same time, it can also be concluded that the type of carbomer used has no significant effect on TC stability.

The progressive decomposition of TC could also be observed from the optical colour changes of the hydrogels during the observation period. In the case of Formulation 1 with a pH value of 8.36, the colour changed very rapidly from light yellow on the first day of observation through dark yellow and orange to dark maroon on day 28. TC in the second formulation with a pH value of 8.04 also showed colour changes, but less intense than was the case with sample 1, which reached a brown colour on day 28. Samples 3 and 4, as well as the second A–C series, maintained the yellow colour of the formulation throughout, with a slightly stronger shade on the last day of analysis. The appearance of a dark maroon colour in samples 1 and 2 is related to the alkaline pH of these preparations, as confirmed by Davies’ studies evaluating the formation of quinones in alkaline environments [27]. 

The second consideration analysed in this study was the activity of the developed formulations against the components of model human skin sebum. Alcoholamines, such as AMPD, TRIS, MEA or TEOA, are agents widely used in cosmetics and pharmaceuticals. They are used as preservatives, foaming agents, emulsifiers and also as substances that stabilize the pH of a preparation. They are used to obtain creams, shampoos, washing preparations and others. They are also often used as gelling agents in polymer preparations [34,35,36]. In an earlier research, Musial and Kubis demonstrated another interesting feature of alcoholamines. They evaluated the activity of these alkaline substances against model sebum components such as stearic acid [20,21,22]. This action is manifested by the penetration of alcoholamine deep into the sebum layer and the appearance of a bright, almost white layer of products. An alcoholamine (a weak base) reacts with weak stearic acid, which leads to a saponification reaction and the formation of amine soap according to Equation (1).

The resulting amine soap, due to the hydroxyl groups in the alcoholamine molecule, binds to the water present in the environment so that an O/W emulsion is formed and the mass is loosened. This phenomenon may facilitate the process of washing out sebaceous accumulations from the hair follicles. Thanks to the effective cleansing of hair follicles with the sebum layer, it is possible to remove the bacteria residing in it that contribute to exacerbations of acne disease, as well as to facilitate the access of therapeutic substances to the interior of the glandular hair unit.

TRIS alcoholamine, which exhibits high activity against model sebum components, was used in this study [19]. Activity against model sebum was analysed for all prepared formulations and the highest activity was shown for sample 1. The composition of this formulation and the excess of free, unbound polymer alcoholamine present in it affected the activity of this hydrogel with model sebum components. The layer of saponification reaction products reached a depth of 1.06 mm after 72 h. For Formulation 2, the polymer content was higher and was 1.20 g, which noticeably affected the activity of this formulation with the sebum layer. The depth of the product layer was less than in sample 1, reaching a depth of 0.69 mm after 72 h. For Formulation 3, the carbomer content was 1.60 g, and the activity of this hydrogel was even lower. After 24 h, the product layer was significantly thin at 0.21 mm, finally reaching a depth of 0.41 mm at the 72nd hour of observation. Despite the equal content of alcoholamines in each hydrogel, noticeable differences in activity could be observed due to the mutual ratio of the components used. Increasing the carbomer content in successive formulations led to a marked decrease in the activity of the formulation and a gradual decrease in the pH value. The large excess of carbomer used in Formulation 4 resulted in complete association of TRIS with polyacrylic acid, as manifested by the formulation’s lack of activity against model sebum components and a decrease pH value of 5.0. In the case of the A–C formulation, the ratio of TRIS to carbomers was 1:1, and the pH of the systems oscillated around 6.35. This content of components did not provide the A–C series hydrogels with activity against model sebum. After 72 h, there was no appearance of at least a minor layer of TRIS reaction products with stearic acid. The type of carbomer used does not affect the activity of the whole system towards the components of the model sebum.

The high amount of alcoholamine unbound to polyacrylic acid in Formulation 1 resulted in a high pH value of 8.36 for the entire system. This value adversely affected the stability of the main component, leading to its rapid degradation. At the same time, it was a feature that promoted the activity of the gel against the components of the model sebum. For Formulation 2, the pH of the system was slightly above 8, which increased the stability of the antibiotic and inhibited the rate of its degradation over 28 days. At the same time, it reduced its activity against the components of the artificial sebum. Formulation 3 proved to be the most advantageous system, in which both the stability of tetracycline hydrochloride and activity against model sebum components were maintained. Formulation 4, in which an excess of polymer was introduced and the pH value of the system was 5.0, showed no activity against model sebum, similar to that of Formulations A–C. These formulations provided high stability of tetracycline hydrochloride without achieving reactivity with sebum components. 

The relative proportions of the components for Formulations 1–4 significantly affected the viscosity of the system. Formulation 1 had the lowest viscosity, oscillating around 20,500 cP. The large excess of polymer in hydrogel 4 caused the viscosity value to almost double compared to Formulation 1 to a value of more than 38,000 cP. Excessively high viscosity is not beneficial for a pharmaceutical preparation. It can adversely affect the release of the drug product from the system, slowing down the optimal therapeutic effect. It can also cause difficulty in distribution on the skin and leave a sticky film on the skin. For Formulations 2 and 3, intermediate values of about 25,000 cP and 29,000 cP were obtained. For Formulations A–C, the viscosity values presented slight differences. For formulation B, which contained Carbopol ETD 2020 NF in its formulation, the viscosity value was the lowest at about 23,700 cP. The viscosity of hydrogel A, containing Carbopol Ultrez 10 NF, was 27,700 cP, while the viscosity of gel C, with Carbopol 980 NF in its formulation, was about 29,500 cP. 

The mutual proportions of the components of the tested formulations and the type of polymers selected can allow the design of a formulation with the most optimal physicochemical properties, while ensuring the maintenance of the active substance tetracycline hydrochloride. Appropriate proportions of polymers in relation to the alcoholamine used make it possible to obtain a system with an adequate pH value while maintaining activity against sebum components. Depending on the necessity and location of application, the type of carbomer can be selected so as to obtain the most desirable viscosity of the treatment formulation.

## 4. Materials and Methods

### 4.1. Reagents

Carbopol 980 NF, an allyl pentaerythritol crosslinked polyacrylic acid homopolymer with a molecular weight as high as 4.5 billion Daltons, Carbopol Ultrez 10 NF, polyallyl pentaerythritol-crosslinked polyacrylic acid homopolymer and Carbopol ETD 2020 NF (which is crosslinked polyacrylic acid interpolymer with a molecular weight close to 4.5 billion Daltons) (Lubrizol, Wickliffe, OH, USA), TRIS (Sigma Aldrich, Poznan, Poland), TC (Sigma Aldrich, Poznan, Poland) and demineralized, bi-distilled water were used to obtain hydrogel formulations [37,38]. Model skin sebum was obtained using triglycerides of animal origin (Fagron, Warsaw, Poland), lanolin (Fagron, Warsaw, Poland), stearic acid (Sigma Aldrich, Poznan, Poland), squalene (Sigma Aldrich, Poznan, Poland) and cholesterol (Sigma Aldrich, Poznan, Poland).

### 4.2. Preparation of Hydrogels

Two groups of formulations were developed to assess the effect of hydrogel components on the stability of the antibiotic contained therein. In the first group, hydrogels 1–4, the effect of the mutual proportions of crucial components on TC stability was evaluated. In the second group, hydrogels A–C, the effect of the type of polymer used on the antibiotic contained in the formulation was analysed.

All hydrogel formulations were prepared with a weight of 100 g. After preparing hydrogels containing appropriate amounts of alcoholamine, carbomer and water, they were conditioned in a fridge at 5 °C for 24 h. After this time, 0.2 g of TC dissolved in 4 g of water was added to each hydrogel. To achieve proper homogenization, each formulation was mixed using an Alpina MR500 pharmacy mixer (Alpina Polska Sp. z o.o., Konin, Poland) for a period of 20 min at the lowest speed of 60–90 RPM to avoid aeration. The compositions of the various formulations are shown in Table 2.

All preparations were stored in a fridge at 4 °C in white, opaque containers throughout the study period.

### 4.3. pH Measurements

Due to the different amounts of polymer used, Formulations 1–4 differed in their pH values. For the A–C formulations, the pH values were similar. The pH value of each hydrogel was measured five times using a pH meter (CPC-505, accuracy up to ±0.002 pH, Elmetron Sp.j., Zabrze, Poland) and a pH electrode (IJ44At HT, Elmetron Sp.j., Zabrze, Poland) for hydrogels 1–4 and a pH electrode (ERH-11S, Elmetron Sp.j., Zabrze, Poland) for hydrogels A–C. 

### 4.4. HPLC Analysis of Formulations

#### 4.4.1. Sample Preparation

For each formulation, 0.5 g of gel was taken and dissolved in 99.5 g of distilled water. The samples were stirred without heating for 20 min on a magnetic stirrer (Arex Digital Pro) (Velp Scientifica, Usmate (MB), Italy) at 900 RPM until completely dissolved in water. An amount of 1 ml of sample was taken from the obtained solutions; five times for Formulations 1–4 and eight times for Formulations A–C. The samples were analysed by HPLC. Samples were taken once a week at equal time intervals for a period of 28 days.

#### 4.4.2. Chromatographic Analysis

HPLC analysis was performed on a Thermo Scientific Dionex UltiMate 3000 instrument (Dionex Corporation, Sunnyvale, CA, USA) equipped with an LPG-3400SD pump, a TCC-3000SD column oven, a DAD-3000 detector and a WPS-3000TSL autosampler. Chromatographic separations for hydrogels 1–4 were performed using an RP-18 LiChroCART column, 125 mm × 3 mm, 5 µm (Merck, Darmstadt, Germany) at 40 °C. The mobile phase was composed of 0.1% formic acid in water (A) and 0.1% formic acid in acetonitrile (B). The flow rate was 1.0 mL/min with the subsequent gradient elution: starting with 7% mobile phase B and maintaining for 0.5 min, reaching 50% in 4 min and 95% in 4.25 min and maintaining for 1 min. From 5.25 min, the gradient returned to 7% mobile phase B and stopped after 6.5 min at this concentration. The retention time was 3.34 min. Aqueous solutions of commercial TC at different concentrations were used to develop a calibration curve (y = 0.2625x − 0.0771, R^2^ = 0.998). 

An RP-18 LiChroCART column, 125 mm × 4 mm, 5 µm (Merck, Darmstadt, Germany) was used to analyse samples of hydrogels A–C. As with previous formulations, chromatographic separation was performed at 40 °C with the same mobile phase composition. The flow rate was 1.0 mL/min with the following gradient elution: starting with 7% mobile phase B and maintaining for 0.5 min, reaching 50% in 4 min and 95% in 5 min and maintaining for 2 min. From 7 min, the gradient returned to 7% mobile phase B and stopped after 9 min at this concentration. The retention time was 4.14 min. For Formulations A–C, commercial tetracycline hydrochloride was also used to determine the calibration curve (y = 0.30693x − 0.15451, R^2^ = 0.998).

For all samples tested, the injection volume was 10 µL and detection was performed at 280 nm.

### 4.5. Macroscopic Observations of the Colour Changes of the Formulations

Macroscopic observations of any visual changes in the test formulations were carried out throughout the drug stability analysis. Colour changes were evaluated under identical lighting conditions using a Sony digital camera DSC-HX300, 20.1–24.3 MPix, 50× optical zoom (Sony, Tokio, Japan) and a 12 MPix iPhone 6s (Apple, Cupertino, CA, USA). 

### 4.6. Artificial Skin Sebum Formulation

The composition of the natural skin sebum has been widely described in the medical literature. It is composed primarily of triglycerides and unsaturated fatty acids, waxes, squalene, cholesterol and its esters [39,40]. We formulated the composition of the model sebum based on our previous research and the available literature [19,21,22]. The artificial sebum developed by Musial and Kubis was found by other researchers to be one of the best, reflecting the lipid composition of natural sebum [41]. All components are shown in Table 3.

All components of the artificial sebum were melted and mixed together in a water bath and allowed to solidify.

### 4.7. Optical Evaluation of the Activity of Hydrogel Formulations against Model Sebum Components

Scaled PVC tubes with an inner diameter of 4 mm were used to evaluate the activity of hydrogel formulations against model sebum components. These tubes acted as a hair follicle model. The scaled pipes were filled with liquid sebum to a certain level and the liquid was allowed to solidify. After the sebum solidified, 0.5 mL of hydrogel preparations were placed on its surface, and the ends of the tubes were plugged with a plastic compound to prevent evaporation of water from the hydrogel. Activity observations were conducted for 72 h at 24 h intervals. Six measurements were taken for each recipe, so the average value of the product layer depth and standard deviation (SD) could be determined. The depth of the reactive sebum layer was determined using a digital measuring device (digital calliper Powerfix IAN Z22855) with an accuracy of 0–100 mm ± 0.02 mm. In addition, digital macro photographs were also taken using a Samsung A53 camera, Macro 5 Mpx, 1.12 µm, FF and f/2.4.

### 4.8. Measurement of Viscosity of Hydrogel Preparations

The viscosity of all hydrogels was measured using a Brookfield rotational viscometer (Middleboro, MA, USA). For Formulations 1–4, spindle 6 and a speed of 50 RPM were used. For hydrogels A–C, a spindle 5 operating at 20 rpm was used. Each measurement was repeated five times so that the average value of hydrogel viscosity and standard deviation (SD) could be calculated.

## 5. Conclusions

The development of an effective method of introducing active substances deep into the hair follicles is an extremely important issue that can significantly impact the effectiveness of topical antimicrobial therapies.

During the design of a therapeutic preparation containing tetracycline hydrochloride in its composition, the mutual proportions of the individual components of the formulation are extremely important both for stability of the antibiotic and for the cleansing and swelling of the sebum layers. The optimal recorded pH of the hydrophilic preparations with the antibiotic is 7.6, and a noticeable swelling activity was still observed in the applied model. An excessively alkaline environment led to rapid degradation of the active ingredient in Formulation 1, which after a week retained about 45% of its original content, while on the last day of observation, the TC concentration decreased to below 10%. The acidic pH of the formulations ensured the high stability of the antibiotic; however, the hydrogels showed no saponification effect with stearic acid in the model sebum.

Proper selection of the mutual proportions of the hydrogel components will allow the design of formulations that show high efficacy in cleansing the hair follicles, followed by effective incorporation of antimicrobial agents into the infection area. Prospective studies may enable the development of formulations with stable and effective composition, which in the future may lead to shorter, as well as more effective, topical antimicrobial therapies.

## Figures and Tables

**Table 1:** pH values.

Formulation	1	2	3	4	A	B	C
pH value	8.36	8.04	7.60	5.00	6.40	6.34	6.30

**Table 2:** Composition of the studied hydrogel preparations.

Formulation	TC (g)	Water (g)	TRIS (g)	Carbopol 980 NF (g)	Carbopol ETD 2020 NF (g)	Carbopol Ultrez 10 NF (g)
1	0.20	96.20	2.70	0.90	-	-
2	0.20	95.90	2.70	1.20	-	-
3	0.20	95.50	2.70	1.60	-	-
4	0.20	93.40	2.70	3.70	-	-
A	0.20	98.80	0.50	-	-	0.50
B	0.20	98.80	0.50	-	0.50	-
C	0.20	98.80	0.50	0.50	-	-

**Table 3:** Components of the artificial skin sebum.

Components	Artificial Skin Sebum
Pork lard as triglycerides	34%
Stearic acid as a free fatty acids	24%
Lanolin as waxes	26%
Squalene	12%
Cholesterol	4%

## Data Availability

Not applicable.

## References

[1] Gollnick H., Cunliffe W., Berson D., Dreno B., Finlay A., Leyden J.J., Shalita A.R., Thiboutot D. (2003). Management of Acne: A Report from a Global Alliance to Improve Outcomes in Acne. J. Am. Acad. Derm..

[2] Bhate K., Williams H.C. (2013). Epidemiology of Acne Vulgaris. Br. J. Dermatol..

[3] Brown S.K., Shalita A.R. (1998). Acne Vulgaris. Lancet.

[4] Katsambas A., Papakonstantinou A. (2004). Acne: Systemic Treatment. Clin. Derm..

[5] Simonart T., Dramaix M., de Maertelaer V. (2008). Efficacy of Tetracyclines in the Treatment of Acne Vulgaris: A Review. Br. J. Dermatol..

[6] Leyden J.J. (2001). Current Issues in Antimicrobial Therapy for the Treatment of Acne. J. Eur. Acad. Dermatol. Venereol..

[7] Nishijima S., Kurokawa I., Katoh N., Watanabe K. (2000). The Bacteriology of Acne Vulgaris and Antimicrobial Susceptibility of Propionibacterium Aches and Staphylococcus Epidermidis Isolated from Acne Lesions. J. Dermatol..

[8] Dréno B., Pécastaings S., Corvec S., Veraldi S., Khammari A., Roques C. (2018). Cutibacterium Acnes (Propionibacterium Acnes) and Acne Vulgaris: A Brief Look at the Latest Updates. J. Eur. Acad. Dermatol. Venereol..

[9] Sapadin A.N., Fleischmajer R. (2006). Tetracyclines: Nonantibiotic Properties and Their Clinical Implications. J. Am. Acad Derm..

[10] Pena A., Carmona A., Barbosa A., Lino C.M., Silveira M.I., Castillo B. (1998). Determination of Tetracycline and Its Major Degradation Products by Liquid Chromatography with Fluorescence Detection. J. Pharm. Biomed. Anal..

[11] Hasan T., Allen M., Cooperman B.S. (1985). Anhydrotetracycline Is a Major Product of Tetracycline Photolysis. J. Org. Chem..

[12] Schlecht K.D., Frank C.W. (1975). Dehydration of Tetracycline. J. Pharm. Sci..

[13] Morrison H., Olack G., Xiao C. (1991). Photochemical and Photophysical Studies of Tetracycline. J. Am. Chem. Soc..

[14] Walton V.C., Howlett M.R., Selzer G.B. (1970). Anhydrotetracycline and 4-epianhydrotetracycline in Market Tetracyclines and Aged Tetracycline Products. J. Pharm. Sci..

[15] Pena A., Palilis L.P., Lino C.M., Silveira M.I., Calokerinos A.C. (2000). Determination of Tetracycline and Its Major Degradation Products by Chemiluminescence. Anal. Chim. Acta..

[16] Benitz K.F., Diermeier H.F. (1964). Renal Toxicity of Tetracycline Degradation Products. Soc. Exp. Biol. Med..

[17] Kelly R.G. (1964). Determination of Anhydrotetracycline and 4-Epianhydrotetracycline in a Tetracycline Mixture. J. Pharm. Sci..

[18] Kostrzębska A., Złocińska A., Musiał W. (2021). Evaluation of the Influence of a Hydrogel Containing AMPD on the Stability of Tetracycline Hydrochloride. Pharmaceutics.

[19] Kostrzębska A., Musiał W. (2020). The Influence of Increasing Concentrations of AMPD on the Efficacy of Its Penetration into a Model Skin Sebum Layer. Pharmaceutics.

[20] Musial W., Kubis A. (2006). Preliminary Evaluation of Interactions between Selected Alcoholamines and Model Skin Sebum Components. Chem. Pharm. Bull. Tokyo.

[21] Musial W., Kubis A. (2003). Preliminary Assessment of Alginic Acid as a Factor Buffering Triethanolamine Interacting with Artificial Skin Sebum. Eur. J. Pharm. Biopharm..

[22] Musial W., Kubis A. (2004). Carbopols as Factors Buffering Triethanolamine Interacting with Artificial Skin Sebum. Polim. Med..

[23] Chopra I., Roberts M. (2001). Tetracycline Antibiotics: Mode of Action, Applications, Molecular Biology, and Epidemiology of Bacterial Resistance. Microbiol. Mol. Biol. Rev..

[24] Durckheimer W. (1975). Tetracyclines: Chemistry, Biochemistry and Structure—Activity Relations. Angew Chem. Int. Ed. Engl..

[25] Wu Y., Fassihi R. (2005). Stability of Metronidazole, Tetracycline HCl and Famotidine Alone and in Combination. Int. J. Pharm..

[26] Younis U.S., Fazel M., Myrdal P.B. (2019). Characterization of Tetracycline Hydrochloride Compounded in a Miracle Mouthwash Formulation. AAPS PharmSciTech.

[27] Davies A.K., McKellar J.F., Phillips G.O., Reid A.G. (1979). Photochemical Oxidation of Tetracycline in Aqueous Solution. J. Chem. Soc. Perkin Trans..

[28] Oka H., Ikai Y., Kawamura N., Yamada M., Harada I.K., Ito S., Suzuki M. (1989). Photodecomposition Products of Tetracycline in Aqueous Solution. J. Agric. Food Chem..

[29] Liang Y., Denton M.B., Bates R.B. (1998). Stability Studies of Tetracycline in Methanol Solution. J. Chromatogr. A.

[30] Drexel R.E., Olack G., Jones C., Santim R., Morrison H., Chmurny G. (1990). Lumitetracycline: A Novel New Tetracycline Photoproduct. J. Org. Chem..

[31] Hoener B.-A., Sokoloski T.D., Mitscher L.A., Malspeis L. (1974). Kinetics of Dehydration of Epitetracycline in Solution. J. Pharm. Sci..

[32] Grobben-Verpoorten A., Dihuidi K., Roets E., Hoogmartens J., Vanderhaeghe H. (1985). Determination of the Stability of Tetracycline Suspensions by High Performance Liquid Chromatography. Pharm. Weekbl. Sci..

[33] Khan N.H., Wera P., Roets E., Hoogmartens J. (1990). Quantitative Analysis of Tetracycline by High Performance Liquid Chromatography on Polystyrene-Divinylbenzene Packing Materials. J. Liq. Chromatogr..

[34] American College of Toxicology (1987). Final Report on the Safety Assessment of Diisopropanolamine, Triisopropanolamine, Isopropanolamine, and Mixed Isopropanolamine. Int. J. Toxicol..

[35] Fiume M.M., Heldreth B., Bergfeld W.F., Belsito D.V., Hill R.A., Klaassen C.D., Liebler D., Marks J.G., Shank R.C., Slaga T.J. (2013). Safety Assessment of Triethanolamine and Triethanolamine-Containing Ingredients as Used in Cosmetics. Int. J. Toxicol..

[36] Becker L.C., Bergfeld W.F., Belsito D.V., Hill R.A., Klaassen C.D., Liebler D.C., Marks J.G., Shank R.C., Slaga T.J., Snyder P.W. (2018). Safety Assessment of Tromethamine, Aminomethyl Propanediol, and Aminoethyl Propanediol as Used in Cosmetics. Int. J. Toxicol..

[37] Varges P.R., Costa C.M., Fonseca B.S., Naccache M.F., de Souza Mendes P.R. (2019). Rheological Characterization of Carbopol^®^ Dispersions in Water and in Water/Glycerol Solutions. Fluids.

[38] Jaworski Z., Spychaj T., Story A., Story G. (2022). Carbomer Microgels as Model Yield-Stress Fluids. Rev. Chem. Eng..

[39] Smith K.R., Thiboutot D.M. (2008). Skin Lipids. Sebaceous Gland Lipids: Friend or Foe?. J. Lipid Res..

[40] Downing D.T., Stewart M.E., Wertz P.W., Colton S.W., Abraham W., Strauss J.S. (1987). Skin Lipids: An Update. J. Investig. Dermatol..

[41] Stefaniak A.B., Harvey C.J. (2006). Dissolution of Materials in Artificial Skin Surface Film Liquids. Toxicol. Vitr..

